# Autism spectrum disorder and low vitamin D at birth: a sibling control study

**DOI:** 10.1186/2040-2392-6-3

**Published:** 2015-01-14

**Authors:** Elisabeth Fernell, Susanne Bejerot, Joakim Westerlund, Carmela Miniscalco, Henry Simila, Darryl Eyles, Christopher Gillberg, Mats B Humble

**Affiliations:** Gillberg Neuropsychiatry Centre, Sahlgrenska Academy, University of Gothenburg, Kungsgatan 12, 411 19 Gothenburg, SE Sweden; Research and Development Centre, Skaraborg’s Hospital, Skövde, Sweden; Department of Clinical Neuroscience, Karolinska Institutet, Stockholm, Sweden; Department of Psychiatry, Faculty of Medicine and Health, Örebro University, Örebro, Sweden; Department of Psychology, University of Stockholm, Stockholm, Sweden; Queensland, Brain Institute, University of Queensland, Brisbane, Australia; Queensland Centre for Mental Health Research, University of Queensland, Brisbane, Australia

**Keywords:** Autism spectrum disorder, Vitamin D, 25-hydroxyvitamin D, Neonatal, Dried blood spots

## Abstract

**Background:**

Insufficient vitamin D activity has attracted increasing interest as a possible underlying risk factor in disorders of the central nervous system, including autism.

**Methods:**

In this study, 25-hydroxyvitamin D (25(OH)D) was analysed in 58 Sweden-born sibling pairs, in which one child had autism spectrum disorder (ASD) and the other did not. The study group consisted of two representative samples; 47 Gothenburg sibling pairs with mixed ethnicities and 11 Stockholm sibling pairs with Somali background. 25(OH)D levels were analysed in the stored dried blood spots taken in the neonatal period for metabolic screening.

**Results:**

The collapsed group of children with ASD had significantly lower vitamin D levels (*M* = 24.0 nM, *SD* = 19.6) as compared with their siblings (*M* = 31.9 nM, *SD* = 27.7), according to a paired samples t-test (*P* = 0.013). The difference was - most likely - not only accounted for by a difference in season of birth between ASD and non-ASD siblings since the mean 25(OH)D levels differed with similar effect size between the sibling pairs born during winter and summer, respectively. All children with African/Middle East background, both the children with ASD and their non-ASD siblings, had vitamin D deficiency.

**Conclusions:**

The findings suggest that low prenatal vitamin D may act as a risk factor for ASD, however, there is a need for replication with larger samples. Future research should study whether or not adequate supplementation of vitamin D to pregnant women might lower the risk for ASD in the offspring.

## Background

Autism spectrum disorders (ASDs) belong to a group of neurodevelopmental conditions that are usually of prenatal origin and, when severe, detected in early childhood. The symptoms comprise a broad range of social-communicative impairments, accompanied by repetitive behaviours and sensory abnormalities. ASD frequently coexists with other cognitive/behavioural/neurological disorders, including intellectual disability, language disorder, attention-deficit/hyperactivity disorder (ADHD) and epilepsy, that is, disorders comprised under the ESSENCE concept (Early Symptomatic Syndromes Eliciting Neurodevelopmental Clinical Examinations) [1].

As regards current studies examining ASD aetiology, genetic factors dominate. However, the importance of interacting environmental factors are increasingly being recognised [2, 3]. Autoimmunity [4, 5], pre- (and some post-)natal infections [6–8], exposure to thalidomide, valproic acid or alcohol during pregnancy [9–11], and older parental age [12] have all been associated with autism. Among perinatally acquired conditions, extreme preterm birth entails an increased risk for neurodevelopmental disorders, including autism [13]. However in spite of extensive research, the relative impact of various pathogenic mechanisms is still insufficiently understood.

Low vitamin D availability has attracted increasing interest as an aetiological factor in disorders of the central nervous system. The initial discovery that rodent brains contain vitamin D receptors, targeted by calcitriol, the hormonally active form of vitamin D, was crucial for this research [14]. The distribution of the vitamin D receptor in human brain has since been established [15]. Stumpf and Privette [16] first suggested that vitamin D deficiency in adults might contribute to the pathogenesis of certain psychiatric disorders, including depression. Later, the negative impact of prenatal vitamin D deficiency on brain development was postulated by McGrath [17], mainly based on epidemiological data. Further research has shown that vitamin D has a role in brain development and function, including neuronal differentiation, axonal connectivity, dopamine ontogeny, immunological modulation and transcriptional control over a large number of genes [18–21]. Prenatal vitamin D deficiency has now been proposed as a risk factor for neurodevelopmental disorders such as schizophrenia [22] and autism [23–27].

Several epidemiological findings indirectly support a possible role for prenatal vitamin D deficiency in autism. For example, dark-skinned immigrants in countries with relative lack of sun have an elevated autism prevalence [28–31]. This is relevant since the synthesis of vitamin D in skin is impaired by melanin [32]. However, the increased risk for autism determined by ethnicity is unrelated to the ethnicity of the father, further implicating the role of fetal environment [33, 34]. Children born to immigrants of Somali origin in Sweden [28, 29] as well as in Minnesota [35], have a very high prevalence of severe ASD with intellectual disability and extreme hyperactivity. Many studies in Europe have documented that dark-skinned immigrants and those wearing concealing clothes have consistently lower 25-hydroxyvitamin D (25(OH)D) levels compared to pale-skinned indigenous populations [36–39], with African girls at highest risk [40].

Season of birth findings constitute another link between autism and vitamin D. In Europe a major proportion of the population have considerably lower 25(OH)D in winter and spring compared to summer and autumn [36, 41]. A number of studies in Sweden, Denmark, UK and north-east USA on season of birth have shown that more children with ASD are born in winter and spring [42, 43], with highest relative rates in March [44, 45] suggesting that lower vitamin D status in the mother during pregnancy may constitute a risk factor [46]. The absence of season of birth effect in Israel [47] and California [48] could be explained by high insolation throughout the year.

A number of studies from different parts of the world, such as Sweden, Egypt and China, report lower 25(OH)D levels in individuals of different ages with ASD compared to controls [49–53]. In none of these studies can reversed causation be excluded, that is, low 25(OHD) levels resulting from ASD symptoms, such as restricted diet and/or more prevalent indoor dwelling. However, to our knowledge there has been no study analysing vitamin D levels in samples taken in the neonatal period.

The aim of the present study was to address the emerging hypothesis that low levels of vitamin D at birth increase the risk for ASD. We examined 25(OH)D levels in dried blood spot samples taken during the neonatal period from children born in Sweden, who later were diagnosed with ASD. We used their siblings without ASD as a joint environmental and genetic control group. Additional contributing factors such as ethnicity, birth order and season of birth were also examined.

## Subjects and methods

### Subjects

A total of 58 sibling pairs were included in the study. Inclusion criteria for the children with ASD were: (1) having been diagnosed with ASD without a known cause according to clinical/neurological assessments, such as Rett syndrome, Fragile X and Tuberous sclerosis during preschool age; (2) having a non-ASD sibling aged 4 years or older; and (3) parents giving consent to assessment of vitamin D status and CMV-infection status (for a separate study) of the ASD and non-ASD affected siblings at birth from dried blood spots. The children were recruited from two different sources, constituting distinct cohorts.

**Figure 1:**
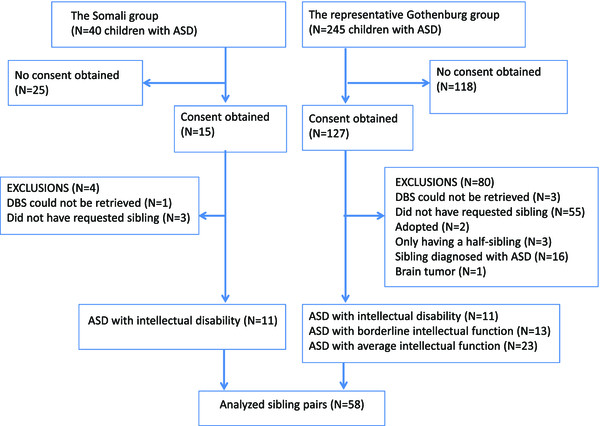
**Participants flow.** ASD, autism spectrum disorder; DBS, dried blood spots.

The first cohort, the Gothenburg catchment area group, consisted of 240 children with ASD born in Sweden between 2005 and 2008 living in the Gothenburg catchment area (approximately 6,000 births/year). The majority of the children in this group had been identified in connection with general population screening at 30 months of age and others had been referred for assessment on clinical grounds. These children had been comprehensively assessed by a multi-professional expert team, specialising in ASD and other neurodevelopmental disorders at the Child Neuropsychiatry Clinic in Gothenburg [54]. Parents of these children were asked to give consent to have their child and his/her non-ASD sibling included in the present study. Consent was provided by 52% of the parents. However, in five cases, the siblings first presumed to be a non-ASD sibling, turned out to also have ASD, increasing the total ASD group to 245 children. A total of 74 children were excluded due to not having a non-ASD sibling, only having a half-sibling or only having a sibling aged younger than 4 years. Another two children had to be excluded because they were adopted, and thus not biologically related to their mother. Three children were excluded since their dried blood spot tests could not be retrieved. One child with a specific brain tumour (ganglioglioma) was also excluded. Of the remaining 47 sibling pairs, 28 had childhood autism, 16 atypical autism and three had Asperger’s syndrome (Figure 1). Two of the children with ASD were siblings and a third sibling served as a control for both. The boy:girl ratio was 8.4:1 (42 boys, 5 girls). With regard to coexisting disorders, 11 also had intellectual disability, 13 had borderline intellectual functioning/developmental delay but not definitely intellectual disability, and 23 had average intellectual function. One child had epilepsy.

The second cohort, the Stockholm Somali group, initially comprised 40 preschool and school children, born in Sweden between 1990 and 2005 by Somali-born parents. This cohort had been assessed at child neuropsychiatric and/or neuropaediatric clinics in Stockholm County by multi-professional expert teams. They were diagnosed with childhood autism combined with intellectual disability and had previously been included in prevalence studies of autism in children with Somali background and their mothers in a vitamin D study [26, 28, 29]. Letters of request to participate in the study were sent to the parents. A total of 15 parents gave consent to the study. However one child could not be identified at the ‘PKU laboratory’, that is, the Centre for Inherited Metabolic Diseases, see below, and another three did not have a sibling aged 4 years or older. Thus, analyses were performed in a total of 11 Somali sibling pairs consisting of one child with ASD and a non-ASD sibling. The boy:girl ratio was 9:1 (9 boys, 1 girl) (Figure 1).

### Ethnicity

The Gothenburg children were of mixed ethnicities; 28 of the mothers were of Swedish origin, five had other non-Scandinavian European origin, four had South American origin, seven had origins in the Middle East and three had origins in East Asia. Ethnic origin was self-reported during interview. All 11 pairs from Stockholm had parents born in Somalia.

Due to the well-known ethnic disparity of vitamin D status, we chose to categorise the participants into three ethnic origin groups: Swedish, Miscellaneous (non-Scandinavian Europe, South America and East Asia) and African/Middle East.

## Methods

### Stored dried blood spots

In Sweden, all newborns are tested for certain neuro-metabolic disorders. Peripheral blood (50 μL) is collected onto a filter paper and then sent to the ‘PKU laboratory’ at the Centre for Inherited Metabolic Diseases, the Karolinska University Hospital in Stockholm where they are stored at a temperature between +4 and +8°C, and kept available for future purposes. The samples taken from the children with ASD and their siblings were retrospectively identified through their mothers’ referral numbers to the laboratory. Each identified dried filter paper was then picked out from its storage box and a 3.2 mm punch was sourced. All dried blood samples were analysed at the Queensland Brain Institute, University of Queensland, Brisbane, Australia for 25(OH)D.

### Vitamin D analyses

25(OH)D can exist in two forms: 25-hydroxyvitamin D_3_ (25(OH)D_3_) and 25-hydroxyvitamin D_2_ (25(OH)D_2_); the latter can only be obtained from dietary sources and supplements. Both 25(OH)D_3_ and 25(OH)D_2_ were measured in the dried blood spots by using a highly sensitive liquid chromatography-tandem mass spectroscopy assay validated for dried blood spots [55]. This laboratory participates in the Vitamin D External Quality Assessment Scheme.

25(OH)D_3_ and 25(OH)D_2_ are highly protein-bound steroids that are completely excluded from erythrocytes [56]. Therefore, to make these results in whole blood comparable with existing studies in sera, 25(OH)D_3_ and 25(OH)D_2_ concentrations were summed and reported as adjusted serum 25(OH)D concentrations [55]. This required a correction based on a standard neonatal capillary hematocrit of 0.61 [57]. In the present study 25(OH)D_2_ was present in only three of the 117 samples. Therefore all data are reported as total 25(OH)D. The laboratory investigators were blind to diagnosis.

### Ethics

The study was approved at the Ethics Committees at the University of Gothenburg and at Karolinska Institutet.

### Statistics

Since the data were non-normally distributed, several methods were used to circumvent this. For the comparisons between cases and sibling controls we used paired t-tests, confirming the results with the boot strap method. We checked the results by means of the Wilcoxon paired sample test and by square root transformation of 25(OH)D values for use in t-tests, obtaining essentially similar results (data not shown). Otherwise, basic statistics were applied. Given our main hypothesis of lower vitamin D status in ASD compared to controls, a case could be made for one-tailed tests, however, our results are conservatively reported as two-tailed with probabilities <0.05 accepted as significant. For the bootstrap method, SPSS was used; all other calculations were performed with Statistica 64, version 10, StatSoft Inc.

Season of birth (winter: December-February; spring: March-May; summer: June-August and autumn: September-November) was assigned for each individual. Because analysis of pairs born during the same season resulted in extremely small sample sizes, we used semesters of birth instead (winter: October-March; summer: April-September) in accordance with a previous Swedish study [58].

## Results

### 25-hydroxyvitamin D related to cohort, ethnicity and birth order in the total sample

**Figure 2:**
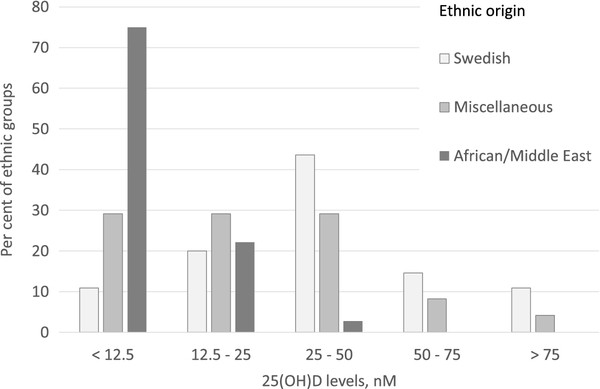
**Distribution of 25(OH)D levels in the total group of children (ASD and non-ASD) with different ethnic origin.**

Children to parents of non-Scandinavian ethnicities had lower mean 25(OH)D levels than children to Swedish parents. The lowest 25(OH)D levels were found in children with African and Middle East origin. The mean 25(OH)D level was 40.3 nM, *SD* = 24.9 in the children with Swedish origin; 31.9 nM, *SD* = 24.9 in children with other European (non-Scandinavian) origin; 25.6 nM, *SD* = 24.2 in children with East Asian origin; 22.4 nM, *SD* = 11.8 in children with the South American origin; 11.5 nM, *SD* = 7.1 in children with Middle East origin and lowest, 7.0 nM, *SD* = 5.0 in children with Somali origin. The distribution of 25(OH)D levels among the Swedish, Miscellaneous and African/Middle East origin children, respectively, are shown in Figure 2.

Gender distribution and age relations between the sibling pairs in the two cohorts are detailed in Table 1. Out of the pairs, 23 were male-male, five were female-female, 29 were male-female, and one was female-male. Two of the children with ASD were born by the same mother. Age relations in sibling pairs are shown in Table 1. The birth order between the child with ASD and his/her sibling was unrelated to the 25(OH)D levels.

**Table 1:** **Gender and median age difference between the ASD child and his/her sibling**

	Gothenburg catchment area group (47 sibling pairs)	Stockholm Somali group (11 sibling pairs)	Total (58 sibling pairs)
Gender in ASD group: M/F (% males)	42/5 (89%)	10/1 (91%)	52/6 (90%)
Gender in sibling group: M/F (% males)	18/28 (39%)	5/6 (45%)	23/34 (40%)
*Age relation in sibling pairs: n; median difference, years (1st - 3rd quartiles)*			
ASD sibling older	14; 1.8 (1.2-2.1)	7; 3.5 (2.3-4.8)	21; 1.8 (1.5-2.8)
ASD sibling younger	32; 2.4 (1.8-4.2)	4; 2.5 (1.8-3.6)	36; 2.4 (1.8-4.1)
Twins (dizygotic)	1; 0.0 (0.0)	-	1; 0.0 (0.0)

### Comparison of 25(OH)D within sibling pairs (child with ASD and sibling without ASD)

The mean 25(OH)D level was lower in children with ASD (*M* = 24.0 nM, *SD* = 19.6, *n* = 58) than in their siblings (*M* = 31.9 nM, *SD* = 27.7, *n* = 57). The difference in mean 25(OH)D was significant according to a paired samples *t*-test (*t*_57_ = 2.57, *P* = 0.013, *d* = 0.33). Since difference scores were highly skewed (*skewness* = 1.86), SPSS bootstrap for paired samples test was performed using 10,000 bootstrap samples. The bootstrap method confirmed that the difference in mean 25(OH)D was significant (*P* = 0.026, 95% confidence interval for the mean difference: 2.24 - 14.21). Comparisons between the ASD and non-ASD sibling in the three ethnic groups, Swedish, Miscellaneous and African/Middle East, are shown in Table 2. Significant differences were found between the ASD and the non-ASD siblings in the Swedish and Miscellaneous groups, but not in the African/Middle East group.

**Table 2:** **Mean 25(OH)D in the children with ASD compared to his/her sibling, separated by ethnic origin**

Mothers’ ethnic origin	ASD child Mean 25(OH)D, nM (SD)	Non-ASD sibling Mean 25(OH)D, nM (SD)	***t (df)*** ^a^	***P*** ^a^
Swedish, *n* = 28	34.5 (20.1)	46.8 (27.8)	-2.14 (27)	0.042
Miscellaneous, *n* = 12	23.1 (16.5)	31.2 (24.0)	-1.55 (11)	0.015
African/Middle East, *n* = 18	8.4 (5.6)	9.2 (6.9)	-.42 (17)	0.68

^a^Due to skewness, these results are calculated on square root transformed values.

### 25-hydroxyvitamin D in children with ASD related to IQ

There was no relation between IQ and vitamin D level, that is, the group with intellectual disability did not display lower 25(OH)D levels compared to those without.

### Season of birth

In the total sample, the difference in season of birth between children with ASD and their siblings was not significant (χ^2^ = 5.5 *DF* = 3, *P* = 0.090, *n* = 115), although 34% of all children with ASD and only 19% of their siblings were born in spring.

In a second analysis considering the different ethnic groups, we joined the sibling pairs into only two groups, due to the small sample sizes. In the Swedish + Miscellaneous group the season of birth of children with ASD differed significantly from that of their non-ASD siblings (χ^2^ = 8.9, *DF* = 3, *P* = 0.030, *n* = 80), as 38% versus 18% were born in spring and 10% versus 35% in summer. However, in the sibling pairs with African/Middle East origin no such season of birth difference emerged (χ^2^ = 1.5, *DF* = 3, *P* = 0.69, *n* = 36).

To examine if the lower level of 25(OH)D in children with ASD as compared to the level in their non-ASD siblings correlated with season of birth, pairs for which both siblings were born during the same six months (Nov-Apr = Winter, *n* = 13 and May-Oct = Summer, *n* = 14) were selected. Both for siblings born during the winter months and for siblings born during the summer months, the mean 25(OH)D level was lower for children with ASD than for their non-ASD siblings, with similar effect size. For pairs of children born during the winter months, the mean 25(OH)D level for children with ASD was *M* = 23.7 nM (*SD* = 10.7, *n* = 13) and for their non-ASD siblings the mean 25(OH)D level was *M* = 26.7 nM (*SD* = 17.4, *n* = 13): *t*_12_ = 0.59, *P* = 0.563, *d* = 0.21). For pairs of children born during the summer months, the mean 25(OH)D level for children with ASD was *M* = 32.4 nM (*SD* = 21.3, *n =* 14) and for their non-ASD siblings the mean 25(OH)D level was *M* = 39.4 nM (*SD* = 24.4, *n* = 14): *t*_13_ = 1.36, *P* = 0.196, *d* = 0.31).

## Discussion

Children with ASD had had lower 25(OH)D levels at birth compared to their non-ASD siblings which most likely was not accounted for by season of birth effects. The findings were seemingly consistent regardless of ethnicity, IQ level and birth order. The study - to our knowledge - is the first in which vitamin D levels in dried blood spots, from the newborn period, have been analysed in samples of children with ASD and in their non-ASD siblings, thereby controlling for shared environmental and genetic causes for ASD. The participants were recruited from two distinct cohorts of Swedish born children with ASD; one drawn from a representative group of children with ASD, living in Gothenburg, the other consisting of children with ASD and with Somali background, living in Stockholm and representing high risk for vitamin D deficiency.

### Ethnicity

Children with ASD in the collapsed sample and in the Swedish and Miscellaneous groups had significantly lower 25(OH)D levels at birth compared to their non-ASD siblings. However in the African/Middle East group a startling floor effect prevented any meaningful comparison in 25(OH)D levels between cases and controls. All these siblings had levels corresponding to 20% of the children of Swedish origin. This is in line with other Scandinavian research, showing that Somali born women have the lowest vitamin D levels of all ethnic groups reported in Sweden [37], even lower than in Turkish and Pakistani mothers living in Norway [59]. In a separate study looking at pregnant Somali-born women in Sweden, 25(OH)D levels were below the detection level of that laboratory (10 nM) in one-third of the cases and below 25 nM in 90% of the cases [60]. Accordingly, the Swedish Somali women and women from the Middle East seem particularly vulnerable to low vitamin D levels, affecting the vitamin D status of the offspring, presumably due to both dark skin [32] and covered clothing.

### Seasonal effects

Our finding that more children with ASD of Swedish or other non-African/non-Middle East background were born in the spring than during other seasons - could possibly implicate other aetiological factors than vitamin D levels, such as, for instance, infections. However, the lack of effect of season of sampling (= season of birth in our study) on 25(OH)D levels in our African/Middle East group, all having low vitamin D status throughout the year, has also been reported in women with African/Middle East origin living in Norway [59]. In conjunction with the elevated incidence of ASD in these populations, this may support a role of vitamin D deficiency - rather than other seasonal factors - in the development of autism. Moreover, even in sibling pairs born within the same 6-month period (resulting in very small numbers for comparison), the ASD sibling had consistently lower levels than the control sibling and with similar effect size as in the total group.

### 25(OH)D levels in other studies on ASD participants

Several studies report lower 25(OH)D levels in individuals with ASD compared to controls [49–53]. Our findings of lower vitamin D levels in children with ASD, compared to their siblings, also accord with results from a population-based study from the Faroe Islands, in the North Atlantic Ocean, of vitamin D levels in adolescents and young adults with ASD. Significantly lower 25(OH)D levels were found in the group with ASD, compared to both their siblings without ASD, their parents and also in comparison to a healthy age and gender matched group [46]. Previous findings of low levels of vitamin D could reflect the consequences of ASD per se, that is, the impact of a person’s indoor lifestyle and circumscribed diet. Since we studied newborn children before diagnosis, our results are definitely unrelated to lifestyle and diet of the individual, although the mother’s lifestyle and other environmental factors cannot be ruled out.

### Genetics and 25(OH)D levels

Genetic causes for low 25(OH)D levels have been suggested, related to, for example, synthesising enzymes and transport proteins [61, 62]. The consistently low levels of 25(OH)D found in individuals with ASD, from the neonatal period up to adulthood, thus may be related, not only to environmental factors (UV exposure and diet), but possibly also to genetic predispositions. However, to our knowledge, no 25(OH)D-related gene has been implicated in genomic studies of ASD. On the other hand, a link between genomic actions of vitamin D and serotonergic deviations in ASD has been proposed by Patrick and Ames [63]. According to these authors, low levels of vitamin D may simultaneously cause hypo-activity of the central serotonergic system and hyperactivity of the peripheral, causing hyperserotoninemia, one of the most replicated biomarkers in ASD research [64].

### Strengths and limitations

The participants of this study represent different ethnic backgrounds, IQ levels ranging from intellectual disability to normal intelligence, and highly variable newborn vitamin D levels. This, along with the study design, allowing comparison between siblings, and the thorough assessment of the children with ASD, all constitute strengths of this study. While the children with ASD were investigated for intelligence level and co-morbidity, the sibling controls were not assessed in person, which is a limitation. However, all children with ASD belonging to the catchment area are registered in the patient registry at the Child Neuropsychiatry Clinic in Gothenburg. If a sibling was diagnosed with ASD we would be informed and some siblings were indeed excluded due to ASD after the study was initiated. Nevertheless, we cannot rule out that additional siblings will be diagnosed with ASD at a later stage, as some children with ASD and normal IQ are not identified until adolescence or later. On the other hand, if a child is diagnosed with ASD after the age of 4 years (which was the youngest age of the sibling controls in this study), he/she probably has a less severe disorder than the index child.

The total sample size was small and not representative with regard to sex ratio in the ASD group, since there were only six girls in the total group of 58 children. Concerning this atypical gender ratio, we cannot explain the high male preponderance in our sample, which may constitute a chance finding. Among the healthy siblings the majority were girls. However, no difference in vitamin D levels related to gender has been found in neonates [65]. More children with ASD were younger than their non-ASD sibling, which could be taken to suggest older parents at birth of the child with ASD (a known risk factor for ASD). However, the age difference within the sibling pairs is more likely a consequence of our sample selection principle: we included children with an ASD diagnosis of age 4 years or older and their siblings also at an age of 4 years or older (so as to minimise the risk that the sibling might have a diagnosis of ASD), meaning that really young siblings could not be included in the study.

The high prevalence of migrant mothers in a Swedish born group of children with ASD, could be viewed as a limitation. In the Gothenburg catchment area group, 41% had foreign ethnicity, reflecting the high frequency of ASD among second-generation immigrants of non-Scandinavian origin reported also in previous studies [54, 66]. Similarly, increased rates of ASD were reported from Finland in offspring to migrant mothers [67]. The Finnish study was based on very large numbers drawn from the Finnish Central Population Register suggesting that our limited sample may nevertheless be representative. Also, in a recent study including 7,540 children with autism drawn from a cohort of more than 1.5 million births, non-white American mothers had increased risk for having a child with ASD compared to white American mothers [68]. Foreign-born black mothers were at highest risk of having a child with both severe emotional outbursts and impaired expressive language. This clinical description is remarkably consistent with the clinical picture of the Swedish Somali children with ASD included in our study [28, 29].

In this study we did not examine other possible causes for ASD, such as for instance infections during pregnancy. Infections are more prevalent during late winter months and thus could explain the increased risk for ASD in children born in early spring which coincide with low 25(OH)D levels. However, CMV-infections were examined from the dried blood spots, and no child with ASD in our study group was found to be affected (yet unpublished data). Autoimmunity is another possible cause for ASD, which was not investigated. However, as low vitamin D is suggested to contribute to the pathogenesis of autoimmunity [69], our findings could be relevant in this context as well.

## Conclusion

Our findings of a low vitamin D level in a group of newborn children, who later developed ASD, provide support for the hypothesis that developmental vitamin D deficiency during late pregnancy may carry an increased risk of ASD in the child, along with several other risk factors. Three different independent findings point towards a role for vitamin D in the development of ASD: (1) Increased risk for ASD in offspring of migrants, especially from countries with a dark-skinned population and from cultures where women use covered clothing; (2) low 25(OH)D levels in groups of newborn children, who later have developed ASD, and in groups of children and adults with ASD; and (3) an association between season of birth and ASD, in our study not extending to the high-risk groups of migrants, suggesting that these groups are exposed to suboptimal vitamin D levels the whole year. Although low levels of vitamin D could have a genetic origin and as such be associated with ASD, our study is the first to rule out ASD-related lifestyle mechanisms as explanation for low 25(OH)D levels, since the samples were taken in the newborn period. Future research should include a larger cohort followed prospectively and also study whether or not adequate supplementation of vitamin D to pregnant women might lower the risk for ASD in the offspring.

## Abbreviations

ASD: Autism spectrum disorder
DBS: Dried blood spot.
